# DYSPHAGIA MANAGEMENT CONTRIBUTES TO BURDEN IN CAREGIVERS OF PERSONS WITH DEMENTIA

**DOI:** 10.1093/geroni/igae098.1627

**Published:** 2024-12-31

**Authors:** Samantha Shune, Ashwini Namasivayam-MacDonald

**Affiliations:** University of Oregon, Eugene, Oregon, United States; McMaster University, Hamilton, Ontario, Canada

## Abstract

Providing care for persons with Alzheimer’s disease and related dementias (ADRD) comes with significant physical and psychosocial costs. Crucially underexplored in its contribution to caregiver burden is dysphagia (swallowing impairment), which occurs in up to 93% of persons with ADRD and contributes to increased morbidity and mortality. The current study aimed to quantify dysphagia’s contribution to general burden among family caregivers of individuals with ADRD. A total of 110, predominately female (79.1%), family caregivers were recruited (mean age 55.8±15.9). Multiple measures of dysphagia severity were considered, including caregiver-reported dysphagia severity (Eating Assessment Tool [EAT-10]) and degree of diet modification (International Dysphagia Diet Standardisation Initiative – Functional Diet Scale [IDDSI-FDS]). General caregiver burden was measured via the Zarit Burden Interview (ZBI). To examine which variables were unique predictors of burden, dysphagia-related variables as well as demographic (caregiver age, sex, employment status, education; care recipient relationship, age, sex) and disease variables (caregiver-reported diagnosis, severity, duration) were all entered simultaneously in a multiple regression analysis. Preliminary regression results indicated that higher ZBI scores were associated with caregivers who reported higher IDDSI-FDS scores (β =.26, p =.0364), identified as female (β =.25, p =.0450), were the spouse of the care recipient (β =.45, p =.0125), and reported shorter disease duration (β =.29, p =.0072). Findings highlight that aspects of managing dysphagia may independently contribute to caregiver burden, particularly in the context of continued oral intake. Thus, inquiry about dysphagia status should be considered in disease management.

